# Plant-Microbe Interactions under the Extreme Habitats and Their Potential Applications

**DOI:** 10.3390/microorganisms12030448

**Published:** 2024-02-22

**Authors:** Pragya Tiwari, Subir Kumar Bose, Kyeung-Il Park, Laurent Dufossé, Mireille Fouillaud

**Affiliations:** 1 Department of Horticulture and Life Science, Yeungnam University, Gyeongsan 38541, Republic of Korea; pragyatiwari@ynu.ac.kr; 2 Department of Biotechnology, Meerut Institute of Engineering and Technology, Meerut 250005, India; bbausubir01@gmail.com; 3 Laboratoire CHEMBIOPRO (Chimie et Biotechnologie des Produits Naturels), ESIROI Département Agroalimentaire, Université de La Réunion, 15 Avenue René Cassin, F-97490 Saint-Denis, France; 4 Laboratoire CHEMBIOPRO (Chimie et Biotechnologie des Produits Naturels), Faculté des Sciences et Technologies, Université de La Réunion, 15 Avenue René Cassin, F-97490 Saint-Denis, France; mireille.fouillaud@univ-reunion.fr

**Keywords:** agriculture, biodiversity, drought stress, extremophiles, phytohormones, plant microbiome engineering

## Abstract

Plant-microbe associations define a key interaction and have significant ecological and biotechnological perspectives. In recent times, plant-associated microbes from extreme environments have been extensively explored for their multifaceted benefits to plants and the environment, thereby gaining momentum in global research. Plant-associated extremophiles highlight ubiquitous occurrences, inhabiting extreme habitats and exhibiting enormous diversity. The remarkable capacity of extremophiles to exist in extreme environmental conditions is attributed to the evolution of adaptive mechanisms in these microbes at genetic and physiological levels. In addition, the plant-associated extremophiles have a major impact in promoting plant growth and development and conferring stress tolerance to the host plant, thereby contributing immensely to plant adaptation and survival in extreme conditions. Considering the major impact of plant-associated extremophiles from a socio-economic perspective, the article discusses their significance in emerging biotechnologies with a key focus on their ecological role and dynamic interaction with plants. Through this article, the authors aim to discuss and understand the favorable impact and dynamics of plant-associated extremophiles and their biotechnological utilities.

## 1. Introduction

Global fluctuations in climatic conditions and environment-induced stresses have a key impact on crop yield and productivity. Statistics have suggested that biotic and abiotic stress have a profound effect on agricultural productivity, with more than 60% of land affected by drought, 9% by deficiency of minerals, 57% of land by extreme cold, 15% by acidic soils, and 6% land by saline conditions [1,2]. To survive and adapt to changes in global climatic conditions, plants have evolved multiple, protective mechanisms to tackle these changes [3,4]. Plant-microbe interactions constitute an evolutionarily favored dynamic association forming an integral component of the ecosystem. Microbes thriving in extreme environments possess genetic and physiological features to survive in diverse and extreme environmental conditions [5,6]. Plant-associated microbes are reported from extreme environmental habitats, namely high and low temperatures, increased salinity, high and low pH, and drought conditions, among others [3,7]. The microbes present in extreme environmental niches are known as extremophiles and possess unique properties to grow and survive in such diverse conditions. Moreover, these microbes may thrive in extreme conditions such as hypersalinity (2–5 M NaCl, designated as halophiles), high and low temperatures (60–115 °C—known as thermophiles), (−2–20 °C—designated as psychrophiles) and diverse pH range (<4 acidophiles and >9 alkaliphiles), respectively [8]. The beneficial microbes associated with plants are classified as rhizospheric, epiphytic, or endophytic and demonstrate multi-faceted attributes from ecological and biotechnological perspectives [9,10].

The extremophiles associated with plants are classified as bacteria, archaea, and eukaryotes, and further into different groups, e.g., *Bacteroidetes*, *Ascomycota*, *Basidiomycota*, *Euryarchaeota*, *Firmicutes*, *Actinobacteria*, *Crenarchaeota*, and *Proteobacteria*. As the beneficial associate, the plant-associated extremophiles display multiple ecological and plant growth promotion (PGP) attributes, positively impacting their plant counterparts [11,12,13]. The plant-associated rhizospheric microbes are present in the root zone and key examples include *Paenibacillus*, *Burkholderia*, *Azospirillum*, *Bacillus*, *Methylobacterium*, and *Pseudomonas*, etc. [14,15], while the epiphytic microbes are present in different phyllosphere zones and may tolerate UV radiations and high-temperature conditions (40–55 °C). The representative examples inhabiting the phyllosphere comprise *Agrobacterium*, *Methylobacterium*, *Pantoea*, and *Pseudomonas*, isolated from different crops in moderate and extreme conditions [16]. The endophytic microbes colonize the internal plant tissues and exist in a mutualistic association with plants [17] and the key examples are *Achromobacter*, *Azoarcus*, *Enterobacter*, *Herbaspirillum*, *Burkholderia*, *Klebsiella*, *Nocardioides*, *Pantoea*, and others isolated from different plant species [18,19]. 

The recent era has witnessed the increased recognition of plant-associated microbes in key biotechnological applications including agriculture, ecosystem restoration, and areas of socio-economic concerns [20,21]. Rhizospheric microbes enhance plant tolerance to abiotic stress through various mechanisms, comprising phytohormone production, mineral solubilization, nitrogen fixation, and plant defense against several fungal and bacterial pathogens [14]. Biological nitrogen fixation by microbes is regarded as a key mechanism in the promotion of plant growth and rhizobacteria enhances plant growth by fixing atmospheric nitrogen into nitrate [22]. Moreover, archaea, root endophytic bacteria, and some rhizobacteria produce antimicrobial compounds that function as biocontrol agents against various pathogens [23]. The plant growth-promoting rhizobacteria (PGPR) are documented in extreme habitats and the key members include *Azospirillum* [24], *Pseudomonas* [25], *Enterobacter*, and *Klebsiella* [26,27], *Rhizobium* [26], *Bradyrhizobium* [28], *Bacillus* [29], *Burkholderia* [30], *Micrococcus* [31] and *Frankia* [32], respectively. 

The progress in high-throughput technologies has provided key insights and knowledge about the dynamics of plant-microbe interactions. In addition, metagenomics tools have facilitated the functional characterization of these extremophiles leading to a better understanding of their potential role in maintaining soil health and plant productivity [33,34]. Through this article, the authors aim to discuss and understand the favorable impact and dynamics of plant-associated extremophiles and their biotechnological utilities. 

## 2. Dynamics of Plant-Microbe Interactions in Extreme Habitats

**Figure 1:**
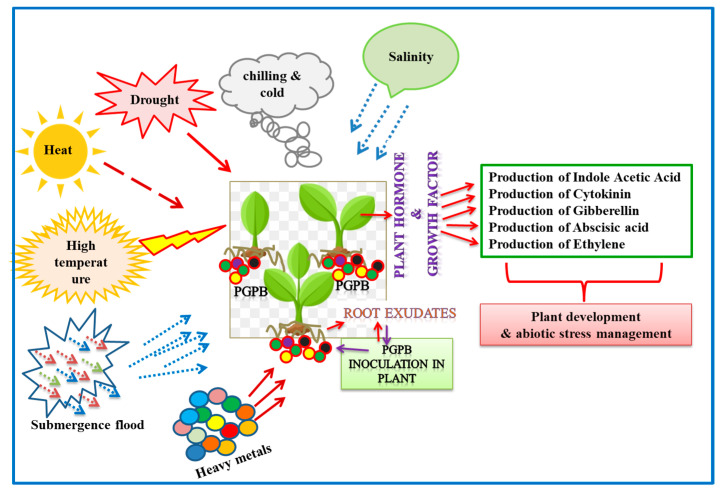
Diagrammatic representation of plant growth-promoting bacteria (endophytic, rhizospheric, and epiphytic) and functional attributes in impacting plant growth, development, and abiotic stress (viz. heat, drought, salinity, chilling, and flood) management.

In the extreme environment, the microbes thrive in the plant vicinity and form symbiotic associations with their plant counterparts. Exhibiting diversity, the microbes are ubiquitous and perform diverse functions, including nutritional uptake, conferring tolerance to environmental stress, and promoting plant growth and development. The existence of microbes in extreme niches raises curiosity to explore the dynamics of symbiotic association, their environmental impact, and biotechnological utilities [35] (Figure 1). 

In extreme habitats, the microbes have evolved distinct genetic and physiological mechanisms to survive and adapt to challenging environmental conditions [8,36]. To get a better understanding, microbes have been isolated and characterized from extreme environments by both culture-dependent and culture-independent techniques [7,37,38] and meta-omics approaches [34]. Omics biology tools have facilitated the elucidation of plant-microbe association at genomics, proteomics, and transcriptomics levels [39,40,41] and provided key insights on the abiotic-induced defense mechanism in plants [42] and socio-economic relevance of PGPB [43], among others. The omics biology-aided analysis of extremophiles ushered in a new era in understanding and decoding these microbes, which were previously unexplored and less understood.

## 3. Diversity and Distribution of Plant-Associated Extremophiles

During the course of evolution, plants co-existed with microbes for millions of years, probably leading to the colonization of early land plants [44]. The microbial diversity was observed in association with plants, namely archaea (*Euryarchaeota*), bacteria (*Acidobacteria*, *Actinobacteria*, *Bacteroidetes*, etc.), and fungi (*Ascomycota* and *Basidiomycota*). The distribution of bacterial species showed variation across different phyla, with *Proteobacteria* being the most dominant followed by *Actinobacteria* [45]. Studies reported the presence of an endophyte (archaea) in association with *Oryza sativa* by culture-independent method [46]. Moreover, the archeal species (isolated from *Euryarchaeota*) were classified into different genera namely *Haloferax*, *Methanobacterium*, *Methanosaeta*, *Methanospirillum*, and *Thermoplasma* [47,48]. 

The microbes inhabiting extreme environments, namely salinity, extreme temperature, extreme pH, and drought, developed special cellular mechanisms for adaptation. Some significant studies on the diversity of microbial extremophiles focussed on alkaliphiles, acidophiles, thermophiles, xerophiles, and psychrophiles [49,50,51]. They are significant in the sense that they have developed distinct features for adaptation to extreme habitats and display biological functions of ecological and biotechnological significance. In a recent perspective, the potential applications of extremophiles in the environment, pharmaceutical, and industrial sectors were extensively reviewed [52]. In addition, extreme environments as biological niches define unique habitats that harbor unique microbes, an area which we believe is worth studying. The diversity and distribution of microbial communities in extreme environments play a critical role in microbial ecology, and diverse species of microbes are isolated from extreme environments, namely high saline conditions (halophiles), high/low temperatures (thermophiles/psychrophiles), acidic/alkaline conditions (acidophiles/alkaliphiles), surviving in earth’s extremely hostile conditions [47]. The microbial diversity in association with the plant is significant for the maintenance of sustainable agriculture. A microbe promotes plant growth and development and provides tolerance to environmental stresses, thereby having a profound effect on plant physiology [53]. The bacterial species classified in *Proteobacteria* are universally distributed in nature, and isolated from crops, such as maize [54], rice [46], wheat [55], and millet [56]. The colonization and distribution of microbial species in a particular plant are governed by the plant genotype and the interacting microbes. These microbes affect plant growth by producing phytohormones and conferring tolerance against pathogens [55]. (Figure 1).

### 3.1. Epiphytic Microbiomes

The above-ground microbial communities (bacteria, fungi, and yeast) in plant association are referred to as the phyllospheric microbes [57]. The aerial zone inhabited by the microbes is known as phyllosphere and the microbes are designated as epiphytes. Mostly, the bacterial communities densely inhabit the leaf surface (epiphytes), however, leaf surface colonization by epiphytes represents a challenging zone for colonization [58,59]. The microbial communities colonizing leaves include different genera of bacteria, algae, filamentous algae, and protozoans. The bacterial species are the most abundant in the phyllosphere and are found in approximately 10^6^ to 10^7^ cells/cm^2^ of the leaf [60]. The epiphytic bacterial communities exhibit size differences among plant species caused by the physical and nutritional condition of the phyllosphere [59]. Moreover, epiphytic microbes have distinct characteristics and show tolerance to high temperatures (40–55 °C) and ultraviolet radiation, being constantly exposed to adverse environmental conditions. Microbes from different phyllosphere zones of crops were reported: *Agrobacterium*, *Methylobacterium*, *Pantoea*, *Bacillus*, *Enterobacter*, and *Pseudomonas* comprising key microbial species in moderate and extreme environmental conditions [59]. Studies have suggested that leaf-colonizing bacteria promote plant growth and confer tolerance to environmental stresses [61]. Recently, sequencing techniques have shown that bacterial communities demonstrating a consistent pattern colonize leaf tissues [62]. The microbes increase plant growth under different abiotic stress conditions. They display different mechanisms of action in plant growth, comprising mineral solubilization, nitrogen fixation, phytohormone production, and siderophores production among others [63]. The epiphytic microbes in the phyllosphere promote nutrient acquisition and uptake by the plants (e.g., facilitating inorganic phosphorous solubilization to soluble form by phosphate solubilizing bacteria) [64]. Other key examples include- phosphorous solubilization by bacterial spp. in *Gossypium herbaceum*, *Brassica nigra*, *Triticum aestivum* [65], phosphorous solubilization by *Pseudomonas* in *A. thaliana* [66], etc. In addition, zinc solubilization is carried out by phyllosphere microbes namely bacterial spp. in *Vigna radiate*, *Triticum aestivum*, *Brassica nigra*, and *Gossypium herbaceum* [65]. Some microbial species, e.g., *Paenibacillus amylolyticus*, *Bacillus mucilagenosus*, and *Psychrobacter fozii* solubilize potassium in the phyllosphere to make it readily available to the plants [55]. Another mechanism in the nutrient acquisition by phyllosphere microbes comprises siderophore production, significant examples include *Pseudozyma aphidis* JYC356 in *Drosera spatulata* Lab. and *Prunus armeniaca* [67], *Bacillus* in *A. thaliana* and *Lycopersicon esculentum* [66], etc. while the uptake of copper, zinc, and sulfur by phyllosphere microflora is also documented [68].

### 3.2. Endophytic Microbiomes

Endophytes constitute bacterial or fungal microorganisms, inhabiting inter/intracellular spaces within plant tissues, and present in almost all plant species. Since plants restrict the growth of endophytes, endophytes evolve mechanisms for adaptation to the environment [69,70], including the production of metabolites for plant growth and development [9,10,11]. Endophytes have been isolated from different plant parts namely meristem, leaves [71], seeds [72], roots [38], and stem, among other tissues. However, studies have suggested that isolation of some endophytes is difficult, and different methods have been employed, namely plant tissue culture on suitable media [72] and endophyte isolation using surface sterilization of ground tissue extract [73]. The fungal endophytes from plants and algae are classified in *Ascomycetes*, while very few reports on *Basidiomycetes* are available [74]. Some of the microorganisms, namely *Penicillium glandicola*, *Acremonium terricola*, and *Phoma tropica*, were classified as fungal endophytes [75]. The endophytic microbial communities comprised of *Achromobacter*, *Azoarcus*, *Burkholderia*, *Enterobacter*, *Gluconoacetobacter*, *Herbaspirillum*, etc. were isolated from different plant species [17,18]. Plant endophyte associations have important ecological and biotechnological attributes [76,77,78]. 

### 3.3. Rhizospheric Microbiomes

The rhizospheric microbial communities present in extreme environments, i.e., drought, salinity, and acidity/alkalinity have developed adaptive mechanisms for survival and are characterized [26,79]. The microbial communities belonging to archaeal phyla *Euryarchaeota* and *Crenarchaeota* and bacterial phyla *Actinobacteria*, *Bacteroidetes*, *Proteobacteria*, and *Firmicutes*) were predominantly present in plant rhizosphere, in extreme habitats [80]. The *Proteobacteria* consist of *α*/*β*/*γ*/*δ*-*Proteobacteria*, found in close association with most of the crop plants. The agriculturally significant species comprise of *α*-*Proteobacteria* which requires low nutrients for growth and induces nitrogen fixation in plant symbiosis. The *β*-*Proteobacteria* have a high metabolic rate and the *γ*-*Proteobacteria* define the largest class, including *Azotobacter* and *Pseudomonas*. The genus *Azospirillum* is closely associated with crop plants namely *Amaranthus*, sorghum, sugarcane, maize, and ryegrass, demonstrating symbiotic nitrogen fixation [81]. Moreover, the distinct examples of rhizospheric microbial communities include *Azospirillum*, *Arthrobacter*, *Burkholderia*, *Bacillus*, *Paenibacillus*, *Burkholderia*, *Pseudomonas*, etc. [15]. The PGPR influences plant growth by stimulating root branches, enhancing the availability of nutrients, and plant protection against pathogens [82]. Such protective mechanisms induced by rhizobacteria may lead to the colonization of nutrient-deficient soils by the plant and the secretion of volatile compounds [83]. Furthermore, mineral solubilization bacteria demonstrate the potential to solubilize aluminum, potassium, phosphorous, and silicon into soluble forms [55]. As more significant studies deciphered the cross-talk between different rhizospheric communities, an understanding of the plant-growth-promoting mechanism became clear. The diversity of the microbial communities associated with the crops is important for maintaining agricultural sustainability. The root exudates from the plant contain diverse substances namely vitamins, organic acids, sugars, amino acids, and antimicrobials which attract rhizospheric fungal communities. The fungal communities derive nutrition from these compounds and promote plant growth by nutrient uptake [84]. The key fungal species from the plant rhizosphere comprise- *Aspergillus* sp., *Eupeniccilium* sp., *Leptosphaerulina* sp. [85], *A. terreus*, *A. luchuensis* [86], *Chrysonilia sitophila* [87], *Aspergillus awamori* [88], *Trichoderma* sp. [89], and *Penicillium* sp. The members have evolved cellular mechanisms to survive and adapt to extreme conditions. The fungal communities show diverse distribution and survive in very high temperatures (115 °C), designated as thermophilic fungi [90]. The key examples of extremophiles comprise halophilic and halotolerant bacteria (*Micrococcus*, *Halobacillus*, *Pseudomonas*), xerophytes (*Frankia*, *Streptomyces*, *Azotobacter*), acidophiles (PGPB *Methylobacterium*, *Pseudomonas*, *Flavobacterium*), and alkaliphiles (PGP *Sphingomonas*, *Arthrobacter*, *Paenibacillus*) [80]. These rhizospheric microbial communities display differential plant growth promotion mechanisms and play a significant role in the environment. 

## 4. Plant-Microbe Interactions in Extreme Ecological Habitats

The plant and the microbial counterpart impact each other, microbes confer tolerance to plants and improve fitness against environmental stresses while in turn, plants can modulate microbial dynamics, enhancing positive interactions [9]. In addition, the associated microbial communities influence plant response to fluctuating climatic conditions: some key interactions that alter plants’ phenotypic plasticity comprise nitrogen fixation symbiosis, PGPRs, mycorrhizal associations, and fungal endophytes, among others promote plant adaptability and defense response in challenging conditions [8,76]. 

### 4.1. Acidic Environment

Acidophiles comprise microbes that thrive in highly acidic environments, as low as pH < 3. Acidophiles are found in diverse ecological niches, including hydrothermal regions, volcanic areas, deep-sea vents, and the stomachs of animals [91,92]. High acidic conditions are found to adversely affect plant growth, leading to changes in the availability of nutrients and soil pH. The plant counters high acidic conditions by maintaining its internal pH. Diverse rhizospheric microbes inhabit acidic environments: acid-tolerant PGPB including *Acidithiobacillus*, *Flavobacterium*, *Lysinibacillus*, *Pseudomonas*, and *Methylobacterium* were isolated and characterized [93]. The best-characterized acidophiles are classified in Archea and bacterial domains [94]. The microbial communities associated with the crop plants are found to be essential for soil health and crop productivity [95,96] and siderophore production alleviates abiotic stress conditions. Moreover, these siderophores mediate iron uptake and convert Fe^3+^ to Fe^2+^ ions in an acidic environment [97]. The existence of low pH in acidic soils leads to the utilization of PGP microbes for plant growth. Furthermore, acidophiles have biotechnological prospects, in the production of vinegar [98], biomining (extraction of metals from ores by microbes) [99], and biofertilizers usage among other significant ones. 

### 4.2. Alkaline Environment

The microbes from alkaline environments can tolerate high pH (>9) and are known as alkaliphiles. These extremophiles possess genetic and physiological mechanisms to survive in harsh conditions. The rhizospheric zone of the plant colonizing alkaline environments such as *Smallanthus sonchifolius*, *Dichanthium annulatum*, and *Chrysanthemum morifolium* comprises diverse microbes from methane and hydrogen-rich environments [80]. In these alkaline conditions, microbes maintain cytoplasmic pH through protein and enzyme activity. Moreover, the alkaliphilic bacteria have the adaptive mechanism to tolerate a diverse range of pH 6–10, with pH 7–8 being optimum for growth [100]. Several distinct PGP microbes have been reported from alkaline environments and a few key ones comprise *Arthrobacter*, *Curtobacterium*, *Paenibacillus*, and *Sphingomonas* sp. In one of the mechanisms, the phosphorous solubilizing bacteria produce acids and survive at pH 12, thereby maintaining cytoplasmic pH. These microbes possess multifarious PGP attributes and promote plant growth in an alkaline environment [101]. 

### 4.3. Drought Condition 

In adverse environmental conditions, plant adaptation and survival are promoted by AM fungi and PGP microbes [102]. In this regard, the drought-tolerant microbes have evolved to adapt/survive in water-deficit conditions and protect host plants by facilitating nutrient uptake and plant growth. The microbes colonize the rhizospheric zone and employ multiple direct or indirect mechanisms including the production of ACC deaminase, phytohormones (abscisic acid, cytokinins, and IAA), bacterial exopolysaccharides, and induced systemic tolerance [103]. In a study by Naylor et al. [104] drought enriched the abundance of *Actinobacteria* in grasses, specifically *Streptomyces* genus. The ability of *Actinobacteria* to form thick cell walls and spores makes it drought-tolerant and leads to a higher presence in the drought areas [105].

Xu and coworkers [106] showed that drought delays the early development of root microbiome, associated with *Sorghum bicolor*. Drought results in the enrichment of root colonizing monoderms, while increasing production of metabolites and increased function of transporters associated with some metabolites. It was also observed that arbuscular mycorrhiza (AM) and ectomycorrhizal (ECM) fungi have key functions in the mitigation of drought stress, aiding water and nutrient acquisition to the plant host [107,108]. An ECM fungus, *Cenococcum geophilum* is present in the dunes of the savannah and dry woodlands, attributed to the higher tolerance of the fungal species to drought conditions [109].

The PGPR produces phytohormones that promote plant growth in stress conditions, for example, IAA governs the differentiation of vascular tissues, and cell division, and promotes the growth of shoots under drought stress [110]. On the other hand, the ABA hormone mitigates drought stress by increasing the transcription of drought-linked genes [111]. ACC deaminase (from bacteria) hydrolyzes ACC into alphaketobutyrate and ammonia [112]. The PGP and drought-tolerant bacteria enhance water potential, and biomass, thereby minimizing water loss in drought conditions [113]. The rhizospheric microbes produce exopolysaccharides and alleviate drought stress in plants [114]. In *Lycopersicum esculentum*, phytohormone strigolactone production was increased on *Rhizophagus irregularis* colonization and exposure to drought, showing a signaling mechanism of the phytohormone in increasing drought tolerance [115]. The drought-resistant rhizobacteria modulate phytohormones and confer drought tolerance- PGPRs (*Acinetobacter*, *Bacillus thuringiensis*, *Azospirillum*, etc.) synthesize IAA that alters root architecture by augmenting root surface area and root tips, nutrient acquisition, and aid plant in overcoming drought [116,117]. Some bacterial species (*P. putida*, *Azospirillum lipoferum*, etc.) produce gibberellin and augment drought stress in some plants [118]. PGPR produces cytokinins that confer plant resistance to drought via inducing cell division, shoot growth, decreasing root-to-shoot ratio, and stomatal opening, among other mechanisms [119,120]. In this regard, a combinational strategy to combine PGPB and endophytes confers stress tolerance to the host plant. The bacterial species adopt molecular and biochemical mechanisms to adapt to drought conditions [121] for instance, the bacteria *Pseudomonas aeruginosa*, *Alcaligenes faecalis*, and *Proteus penneri* enhance protein content, water content, and sugars in maize [122]. These examples suggest that the association of drought-tolerant extremophiles promotes plant growth, adaptability, and survival under drought conditions.

### 4.4. High Temperature

High-temperature conditions adversely affect plant growth by altering membrane permeability, seed germination, and rate of photosynthesis [123]. The rhizospheric microbes of plants inhabiting extremely high temperatures, *Triticum aestivum*, *Cupressus dupreziana*, and *Sporobolus indicus*, promote plant growth in hot conditions. PGPB increases plant growth through several mechanisms, comprising nitrogen fixation, solubilization of P, and Zn, phytohormone production, HCN, and siderophore production [124]. Due to the ability of PGPB in plant growth, many bacterial genera namely *Arthrobacter*, *Streptomyces*, *Pseudomonas*, and *Staphylococcus* sp. are used as bioformulations for plant growth in high-temperature conditions. In high temperatures, the metabolism and physiology of extremophiles are adversely affected, and the microbial enzymes promote high-temperature acclimatization and protection of cell structure and integrity via increased expression of heat-tolerant proteins [8]. Several fungal species have been isolated from hot habitats and comprise *Talaromyces thermophilus*, *T. byssochlamydoides*, *Malbranchea cinnamomea*, *Aspergillus terreus*, *Myceliophthora fergusii*, *Thermomyces lanuginosus* [125], *Myceliophthora thermophila* [126], *Scytalidium thermophilum* [127], and others [128]. The fungal communities protect the plant by performing several functions, which include P, potassium (K), and Zn solubilization, phytohormone production, and siderophore production for plant adaptation and survival [84,95,96]. In a study, Waqas et al. [129] reported that *Paecilomyces formosus* (an endophytic fungus) plays an important role in plant adaptation to heat stress and the production of secondary metabolites and phytohormones. Furthermore, thermotolerant microbes that efficiently solubilize phosphate act as excellent biofertilizers in agriculture. The key mechanism of microbes which solubilize phosphate convert insoluble phosphorus to a soluble form, improving phosphorous acquisition [130]. Shekhawat and coworkers [131] showed that *Enterobacter* sp. SA187 (a root endophyte), enhanced heat tolerance in plants, mediated by ethylene signaling via histone protein modification in heat stress genes *HSP18.2.* and *APX2*, epigenetic modifications leading to the priming effect. In another study, ethylene signaling positively impacted heat tolerance in rice and tomatoes [132,133]. 

### 4.5. Low Temperature

The microbes inhabiting extremely cold temperatures are designated as psychrotrophic microbes and have prospects in medicine, agriculture, and industries. Microbes from cold habitats are universally present, found in mountain caps, glaciers, frozen lakes, and snow, and in association with plants growing in cold habitats. Diverse microbes were isolated by culture-dependent and culture-independent techniques and classified as *viz.*, *Euryarchaeota*, *Ascomycota*, *Basidiomycota*, *Chlamydiae*, *Cyanobacteria*, *Actinobacteria*, *Chloroflexi*, *Bacteroidetes*, etc. [134]. The microbes inhabiting cold climates define importance in the ecological perspective since a considerable portion of aquatic and terrestrial ecosystems are influenced by cold temperatures. Moreover, cold regions extremophiles have been reported from Antarctica and extreme cold regions of the world. The extremophiles show extreme diversity and novel psychrophilic microbes comprise *Oleispira antarctica* [135], *Flavobacterium frigidarium* [136], *Octadecabacter arcticus* [137], *Sphingomonas glacialis* [138], *Halobacterium lacusprofundi* [139], and *Cellulophaga algicola* [140]. A key application of psychrophiles comprises the production of thermostable enzymes (β-glucosidase, amylase, etc.), antibiotics, and anti-freezing substances of industrial importance [141]. The microbes contribute to plant growth by several mechanisms either by mineral solubilization (K, Zn, P), nitrogen fixation, siderophores production, phytohormone production, or by conferring tolerance to plant pathogens [9].

### 4.6. Saline Condition

Globally, most of the land in agriculture is threatened by the presence of saline conditions that result in poor microbial functions, due to osmotic stress and ion-induced toxicity [103] detrimental to plant growth. Moreover, soil salinity adversely affects the plants including seed germination, uptake of nutrients and water, crop productivity, and ecological balance [142]. Several studies demonstrated the beneficial effect of PGP and endophytic microbes in mitigating the negative effect of salinity in soil [143]. In a key example, the PGPB *Pseudomonas stutzeri*, when inoculated in salt-sensitive and tolerant plants, reduced the adverse effects [144]. Sometimes, inoculation of salt-tolerant bacteria together with AM fungi considerably improves the plant tolerance to salinity stress [145]. Salinity-tolerant microbes adopt several direct and indirect mechanisms to counter salinity stress, and these include the production of phytohormones, mobilization of nutrients, nitrogen fixation, and siderophore production [146]. These microbial mechanisms contribute to root length increase, number of roots, and surface area by uptake of nutrients [147]. Moreover, the major indirect mechanism of salt-tolerant microbes includes resistance to pathogen infection by decreasing their frequency. The microbial exopolysaccharide induces resistance to salinity stress by cations binding thereby limiting its availability to the plant [113]. The rhizobacteria (*Bacillus subtilis* and *Bacillus pumilus*) from soil showed PGPR functions, hydrogen cyanide and ammonia production, IAA production, tolerance to salt stress, and phosphate solubilization [148]. Bano and Fatima [149] showed that PGPB *Pseudomonas* and *Rhizobium* mitigate salinity-induced stress in *Zea mays*. Similarly, *B. pumilus* and *P. pseudoalcaligenes* reduce the activity of superoxide dismutase and lipid peroxidation in salinity-sensitive rice plants [150]. Recently, deZelicourt et al. [151] showed that an ethylene precursor, 2-keto-4-methylthiobutyric acid (KMBA), produced by *Enterobacter* sp. SA187 (from the desert plant *Indigofera argentea*) promoted the growth of alfalfa and *A. thaliana* under salt stress via enhanced K^+^/Na^+^ ratio in roots and shoots and increased expression of KMBA pathway genes [151]. 

### 4.7. Presence of Heavy Metals

The presence of heavy metals in agrosystems has intensified across the globe and the high concentration of heavy metals is toxic and adversely impacts plant growth and functions. The decrease in crop yield affects human health and food demands, cadmium (Cd) and lead (Pb) are the major toxic heavy metals affecting *O. sativa*, a staple food crop. In addition, the accumulation of heavy metals in crops can cause serious health damage. 

In recent times, heavy metal stress alleviation employing microbes is gaining importance, and key examples include *Pseudoalteromonas* sp., *Bacillus*, *Salmonella* sp. [152,153,154]. In heavy metal-stress plants, rhizobacteria produce IAA and elevate plant growth in polluted soil via macro and micronutrient uptake and conferring plant tolerance to heavy metals [117]. An interesting example is *Deinococcus radiodurans*, an extremophile bacterium that occurs in soil [155]. The bacteria have a high concentration of Mn^2+^-metabolite complexes that can scavenge ROS [156]. The inoculation of rice plants with the bacteria releases antioxidants that improve plant tolerance to Pb and Cd stress. Studies have discussed the application of exopolysaccharides from extremophiles in bioremediation, via bioaccumulation of heavy metals [157].

### 4.8. Flooding Condition

In the fluctuating climate scenario, the increase in flood conditions poses havoc for land plants and causes flooding stress. The flooding stress adversely impacts the plants and causes metabolic and physiological changes and alters the plant-associated microbiome. The nature-based solutions to tackle flooding stress utilize living organisms to minimize the effects of climatic fluctuations [158,159]. Ravanbakhsh and coworkers [160] showed that multiple plants inoculated with ACC deaminase-producing bacteria under flooding, improve plant growth by decreasing ethylene synthesis. In another example, Farwell et al. [161] discussed that under flood and nickel stress, inoculation of the canola plant with *P. putida* UW4 increases plant biomass and growth, improving plant adaptation. 

## 5. Biotechnological Applications of Plant Microbiome

Plant microbiome highlights significant biotechnological prospects comprising of decomposer bacteria/fungi that easily decompose plant waste and produce organic manure, biofertilizers that increase agricultural production multiple times, and pathogenic microbes that kill harmful diseases and bacteria, among others. Nowadays, scientists are focusing on organic farming by using and identifying natural microbes mainly PGPB, and creating synthetic communities via molecular biology and modern biotechnologies [162,163] (Table 1).

### 5.1. Plant Growth Promotion 

PGPR colonizes the rhizospheric zone, attracted by plant root secretions composed of different chemical attractants. PGPR as microbial inoculants facilitate mineral acquisition and positively impact plant growth in agro and allied cultivation [181] through upregulation of plant hormones [182], and indirectly through inhibitory effects on soil-borne pathogens [183].

#### 5.1.1. Production of Phytohormones

Phytohormones play an integral role in affecting plant growth dynamics via multiple physiological and biochemical changes in the plant life cycle [184,185]. In the mitigation of biotic and abiotic stresses, PGPB found in the rhizospheric zone secretes many phytohormones and modulates the concentration of specific growth hormones in the plant [182]. In the rhizospheric zone, different rhizosphere colonizing bacteria were shown to produce phytohormones to enhance plant growth [186,187]. Phytohormones are chemical messengers that in small amounts regulate cellular activities, key examples include abscisic acid, cytokinin, auxins, brassinosteroids, and jasmonates, etc. and some are key targets for plant metabolic engineering for conferring abiotic stress tolerance [188,189]. *Cucumis sativus* root secretes vanillic acid and p-coumaric acid which demonstrate differential effects on the soil microbiome. In the study, p-coumaric acid attracts the pathogenic fungal taxa that degrade the p-coumaric acid [190], while vanillic acid promotes the activity of PGPR [191]. The phytohormone, IAA (produced by PGPB in large amounts), directly functions in cell differentiation and division, as well as cell elongation in plants [192]. In a key study, beneficial effects on root elongation and lateral root production in *Z. mays* were observed on *Acinetobacter and Pseudomonas* inoculation. While *R. leguminosarum* improves the early seedling root growth of the non-legume canola and lettuce via cytokinin and IAA production, *Trichoderma* sp. biosynthesize auxins and stimulates plant growth by stress mitigation [193].

Rhizosphere colonizing or endophytic ACC deaminase-producing bacteria alters plant ethylene levels [194]. Abscisic acid (ABA) greatly assists plants in countering environmental stresses and is actively involved in various defense mechanisms. Shahzad et al. [195] investigated the favorable impact of *Bacillus amyloliquefaciens* inoculation in rice on plant growth attributes in salinity conditions. Tiwari et al. [196] demonstrated that *P. putida* was effective in mitigating drought conditions via ABA biosynthesis, in chickpeas. From the rhizospheric soil of grapevines, *Bacillus licheniformis* Rt4M10 and *Pseudomonas fluorescens* Rt6M10 were isolated and demonstrated ABA, IAA, and GA_3_ production. The result showed that ABA concentration increased as compared to control [197]. Furthermore, *Bacillus licheniformis* SA03 in *Chrysanthemum* plants decreased salinity stress via modulating photosynthesis and biochemical mechanisms [198].

Presently, many scientific reports available are related to PGPB’s effect on plant growth and yield enhancement [180,199]. Abiotic stress induces the production of ACC deaminase by PGPB, *Solanum lycopersicum* inoculated with *B. subtilis* demonstrated a significant increase in chlorophyll content and plant biomass. In another report, *Z. mays* showed higher drought tolerance, chlorophyll levels, higher plant biomass, and lower phytohormone levels [200]. Inagaki et al. [201] investigated the beneficial effect of plant inoculation with a bacterial consortium that improved physiological parameters such as chlorophyll content, leaf area, diameter of stem, etc., and increased nitrogen acquisition and uptake by the plant.

#### 5.1.2. Biological Nitrogen Fixation

Nitrogen is a very important element for plant growth since it plays a major role in amino acid synthesis, the key building blocks of proteins, and is a major component of chlorophyll, an important pigment for photosynthesis. It is also found in other important biomolecules, such as nitrogen bases including nucleotides and nucleosides (ATP, GTP, CTP, and TTP, etc.) and nucleic acids. Prokaryotic organisms possess a widespread ability to fix atmospheric nitrogen [202]. PGPB positively impacts plant growth via- direct mechanisms, including multiple processes such as nitrogen fixation, phosphate solubilization, production of siderophore, ammonia, and phytohormones, etc. while the indirect mechanisms comprise antibiotic production, ACC deaminase activity, induced systemic resistance (ISR) among others [203]. 

The bacterial endophyte, Bradyrhizobia in *O. sativa* produces IAA and ACC deaminase and fixes atmospheric nitrogen during symbiosis [204]. The enhanced IAA biosynthesis induces nitrogen fixation in the plant and may be used as a biofertilizer [205]. The isolated nitrogen-fixing bacteria *Azotobacter chroococcum* and *Azospirillum lipoferum* were used as biofertilizers and enhanced growth and essential oil yield in three species of *Mentha* plants [206].

#### 5.1.3. Mineral Solubilization

Plant well-being is greatly influenced by mineral nutrients and during abiotic stress, plants are unable to absorb the minerals and micronutrients severely hampering plant growth and leading to plant disease. The above damages are corrected naturally by microbes that can convert complex forms to simple forms that are easily absorbed by the plants (e.g., siderophores). Siderophores are produced by bacteria, fungi, and plants to facilitate the uptake of iron [207,208] and function as iron chelators (bind iron present in the rhizosphere). 

The poor availability of inorganic phosphate (orthophosphate) in soil hampers crop production [209]. The phosphate solubilizing bacteria converts insoluble inorganic phosphate [210] to soluble forms and improves phosphorous availability for the plant. Joe and coworkers [211] isolated *Acinetobacter* sp. and *Bacillus* sp. from *Phyllanthus amarus* which showed phosphate solubilization and salt tolerance and increased plant growth compared to non-inoculated plants. The mycorrhizal fungi and vesicular-arbuscular mycorrhizal (VAM) fungi are interesting examples of plant growth promoters via forming extensive fine hyphae and improving nutrient acquisition. 

#### 5.1.4. Biocontrol Function

Biocontrol agents secrete biochemical and other substances and inhibit harmful pathogenic bacteria without damaging plants and soil. The plants/crops are disease-affected by reducing crop yields, contamination of food grains, and declining production quality. Multiple PGPB synthesizes salicylic acid that signals systemic acquired resistance (SAR) while PGPB may start to induce systemic resistance (ISR), enhancing plant defense against plant pathogens [180]. PGPBs are key players in disease management, maintain ecological subsistence, and reduce the deleterious effects of chemical fertilizers [212,213]. The representative examples include *Arthrobacter*, *Enterobacter*, *Pseudomonas*, *Rhizobium*, and *Frankia* spp. 

Microorganisms produce antibiotics as important mechanisms to control phytopathogens. While *Pseudomonas* sp. synthesizes diverse compounds namely amphisin, hydrogen cyanide, 2,4-diacetyl phloroglucinol (DAPG), pyrrolnitrin, phenazine, etc. *Streptomyces*, and *Bacillus* sp. produce xanthobaccin, oligomycin A, and kanosamine, with potent antimicrobial functions. In addition, chitinases produced by microbial species inhibit fungal pathogens via degrading fungal mycelium. PGPB produces hydrogen cyanide that increases its antifungal properties [183]. 

### 5.2. Mitigation of Multiple Abiotic Stress

Recent agricultural trials have scientifically validated that PGPRs not only reduce environmental stresses but also increase the production of a variety of crop plants, such as soybeans, mint, rice, barley, and maize [146,214,215,216]. Hormones primarily control the prioritization of signals carried out by protein switches such as kinases, transcription factors (TFs), and G-proteins, according to molecular research (gene expression profiling). Usually, plants focus their physiological resources on abiotic stress adaptation, which renders them prone to biotic stressors such as herbivory and disease [217]. 

The hormone that is primarily involved in the abiotic stress response is ABA. Plants respond to abiotic stress through defense mechanisms activated by ethylene, salicylic acid, or jasmonate [218]. For instance, increasing the generation of ROS to reduce loss during abiotic stress may shield plants from assault by biotrophic diseases, but it also increases their susceptibility to necrotrophic infections. Understanding these intricate interactions between plants and microbes and their dynamics in the context of an abiotic stress response may be aided by using omics techniques.

Microbe-mediated development of abiotic stress responses is often referred to as induced systemic tolerance (IST). Over the past few decades, there has been a lot of research conducted regarding the function that microbes play in helping plants cope with abiotic challenges [219]. Plants experience less abiotic stress thanks to the possible inherent metabolic and genetic capacities of microbes [180,182]. *T. harzianum* function in the rice genotype decreases stress through the overexpression of physiological genes, specifically those encoding aquaporin, dehydrin, and malondialdehyde [175]. The rhizosphere makes up a soil microclimate around the root zone, where the average number of microbes is significantly higher than in the bulk soil. Therefore, it is evident that a variety of nutrients, minerals, and metabolites found in plant roots may play a significant role in drawing microbes to gather and form partnerships with plants. One of the most important things that plants do to facilitate microbial colonization in the rhizosphere is to secrete root exudates.

#### 5.2.1. Heat Stress 

The microbes belonging to different genera including *Azospirillum*, *Achromobacter*, *Variovorax*, *Enterobacter*, *Bacillus*, *Azotobacter*, *Klebsiella*, *Aeromonas,* and *Pseudomonas* promote plant growth under heat conditions [220]. *T. aestivum* inoculated with *Azospirillum brasilence*, and *Bacillus amyloliquefaciens*, under heat stress resulted in reduced regeneration of ROS (reactive oxygen species), pre-activation of heat shock transcription factors, and changes in metabolome [221]. 

#### 5.2.2. Cold Stress 

The freezing (cold) damage is one of the main causes of crop loss [222,223]. It lowers crop production and productivity by slowing down plant growth and development [224,225]. PGPRs are beneficial to many plants as they increase their resistance to various stressors, such as low temperatures. Su et al. [226] showed that *Burkholderia phytofirmans* PsJN decreased the effect of freezing temperatures on *A. thaliana* photosynthesis.

#### 5.2.3. Drought Stress

According to Mittler et al. [227] and Cramer et al. [2], agricultural loss occurs in different crops due to abiotic stress namely water deficit (drought) conditions, and affects 64% of the global land area, respectively. The potential of microbial interactions with the plants has, therefore, multifaceted functions, one of them is adaptation under drought stress. The root fungal endophyte *Piriformospora indica* induces drought tolerance in Chinese cabbage by increasing the levels of antioxidants and improving many physiological parameters [228]. 

#### 5.2.4. Salinity Stress

*T. harzianum* application to increase the oil content in NaCl-affected Indian mustard (*Brassica juncea*), improved nutrient uptake, and improved the accumulation of antioxidants and osmolytes while decreasing NaCl uptake [229]. Concurrent with these results, it was shown that plants treated with *Trichoderma* produced higher levels of monodehydroascorbate reductase. Additionally, research on mutants has verified that *Trichoderma* produces ACC-deaminase, which reduces the effects of salt stress [230]. *Pseudomonas* sp. and *Acinetobacter* sp. have been shown to increase the production of IAA and ACC deaminase in salt-affected soil in oats and barley [231]. *B. phytofirmans* strain PsJN mitigates drought stress in maize [232], wheat [233], and salt stress in *Arabidopsis* [234]. Salt tolerance in rice variety improved in germination under salinity stress via *Pseudomonas* sp. inoculation. Sen and Chandrasekhar [216] reported that *Pseudomonas* sp. can produce exopolysaccharides (EPS) that lead to enhanced tolerance toward salinity stress. Kumar et al. [235] have shown that the inoculation of *Bacillus pumilus* improved rice growth in response to salinity stress. A possible mechanism suggested was that higher expression of ROS scavenging enzyme machinery (in the presence of PGPR) may lead to healthy plant cells and protect them from stress conditions. Palaniyandi et al. [236] showed the alleviation of salt stress and growth promotion by *Streptomyces* sp. strain PGPA39 in ‘Micro-Tom’ tomato plants. The root fungal endophyte *P. indica* induced salt tolerance in barley [237] by increasing the levels of antioxidants and improving many physiological aspects. 

## 6. Plant Microbiome and Ecological Perspective

Soil represents one of the most highly diverse ecosystems on the earth with interacting communities of viruses, bacteria, fungi, archaea, protozoa, and small arthropods. They survive near the plant and are collectively known as the soil and plant microbiome. Microbiota plays an important role in plant growth, development, health, yield, and stress mitigation. The root system of the plant which mainly provides anchorage and uptake of water and nutrients is key to a plant interacting with its surroundings [238]. Natural microbes are found in the rhizosphere, including groups of microbes namely bacteria, fungi, actinomycetes, protozoan, etc. The chemicals present in plant root exudates attract microbes and secretion of these chemicals varies between different plant species, ecotypes [239], and even distinct roots within a plant and include amino acids, aliphatic acids, proteins, sugars, flavonoids, fatty acids, etc. [240]. All of the biochemical secretions may attract and initiate both symbiotic and pathogenic interactions within the rhizosphere [241]. Rasmann et al. [242] showed that maize roots damaged by insects emit the volatile compound (E)-β-caryophyllene, which attracts entomopathogenic nematodes. 

According to Rudrappa et al. [243] *A. thaliana* leaf infections caused by *Pseudomonas syringae pv. tomato* (Pst) bacteria result in malic acid exudation from the roots, which attracts the beneficial *Bacillus subtilis* strain FB17. This rhizobacteria was encouraged to connect to the plant roots by the greater quantities of malic acid, which was followed by the production of biofilms. In a study, Pst leaf infection of *A. thaliana* resulted in higher levels of long-chain organic acids (LCOAs) and amino acids, but decreased levels of sugars and short-chain organic acids (SCOAs) as compared to plants that hadn’t been infected [244]. While these exudates did not directly hinder the pathogen’s growth, they did elevate the expression of *phlA*, a gene implicated in *Pseudomonas fluorescens* production of the antifungal chemical 2, 4-diacetylphloroglucinol (2,4-DAPG). Sometimes microbes secrete chitinase cell wall degrading enzyme chitinase and α-1,3-glucanase [245] which trigger the induction of systemic acquired resistance (SAR) [246]. Among the plant exudates, the indole-derived benzoxazinoids (BXs) have been long implicated in direct plant defense against pests and diseases above and belowground [247]. A study according to Hu et al. [248] revealed that BXs released by maize roots rhizosphere influence the microbiome composition of the next generation of maize plants.

## 7. Concluding Remark and Future Perspective

Plant-associated extremophiles define an important interaction displaying ecological and biotechnological significance/utilities. Plant microbiome research has attracted the attention of various other research disciplines, including botany and plant ecology [249,250], restoration and invasion ecology [251], phytoremediation [252], mathematics and modeling [253,254], and chemistry and natural product discovery [255]. The remarkable capacity of extremophiles to exist in extreme environmental conditions is attributed to the evolution of adaptive mechanisms in these microbes at genetic and physiological levels. The multi-faceted roles extremophiles play in positively impacting their plant counterparts via boosting plant growth, biocontrol mechanisms, conferring biotic/abiotic stress tolerance, and improving plant adaptation in extreme ecological niches, has contributed to the increased recognition and exploration of extremophiles in the present decade. 

The advances in genome sequencing, genetic manipulation, and omics biology have elucidated the intricacies of plant-microbe dynamics, and improved plant adaptability and stress tolerance in challenging climatic conditions. While high-throughput sequencing has unraveled the high genetic variability in the soil microbiome, tools in omics biology e.g., metagenomics offer prospects in the diagnosis of phytopathogens and expanding horizons in plant microbiome studies. Transcriptome analysis based on Next Generation Sequencing (NGS) is a useful technique in deciphering the molecular mechanisms in plant-microbiome interactions. However, the enormous data generated for higher plants makes interpretation difficult. 

Nowadays, crop production is facing many challenges such as climate change, toxic chemical exposure, heavy metals, and demographic development, and it is difficult to address the increasing global food demands. These challenges can be tackled by employing plant-beneficial microbes for sustainable practices. As plant-associated extremophiles have the potential to produce nitrogen, phosphate, and other micro-nutrients in plants, and PGPRs improve plant health, positively impacting crop yield and production, these are frequently used as biofertilizers in agricultural practices [256,257]. In the market, microbe-based biofertilizers are available commercially in many brands as Azo-Green, BaciGold, Custom GP, Custom N_2_, Diegall, Galltrol, Gmax PGPR, Nitromax, Subtilex, Yield Shield, Root Shield Plus WP, and others.

A particularly interesting concept of ‘plant microbiome engineering’ or PME has been emerging, and co-integration with traditional agricultural practices can boost the microbial ecosystem for crop yield and resilience [258]. The manipulation of beneficial plant microbiomes offers an interesting opportunity to promote sustainable agriculture, in the uncertainties of climate change. The fundamental aim of ‘microbiome engineering’ is to promote plant health and functions, supported by omics biology tools, and will bridge the knowledge gaps on factors affecting microbiome assemblage and plant-microbial dynamics. To add value, an optimized phyto-microbiome will reduce soil pollution and enhance sustainable agriculture, leading to ecological subsistence. Although plant-associated extremophiles are recently gaining recognition and have been characterized by diverse ecological niches, their prospects and potential in positively benefiting human lives and their biotechnological utilities define an area needing further research. 

## Figures and Tables

**Table 1:** Ecological and Biotechnological applications of plant-associated extremophiles.

S. No.	Microbial Species	Extreme Habitat	Biotechnological Utilities	Reference
1.	*Bacillus halodurans*	Alkaliphiles	Enzyme production Amylase	[164]
	*Pseudalteromonas* sp. CP76	Halophiles	Proteases	[165]
	*Bacillus subtilis* A-53	Psychrophiles	Cellulases	[166]
	*Bacillus firmus* *Psychrobacter okhotskensis*	Alkaliphiles Psychrophiles	Xylanases Lipases	[167,168]
	*Lactobacillus reuteri*	Halophiles	Glutaminase	[169]
	*Acinetobacter* sp.	Psychrophiles	Esterase	[170]
	*Thermoplasma acidophilum*	Thermophiles	Chitinase	[171]
2.	*Pseudomonas rhodesiae* *Bacillus amyloliquefaciens*	Psychrophiles	Plant growth promotion	[172]
	*Pseudomonas fluorescens* IARI-HHS1-4		Biological nitrogen fixation	[172]
	*Rahnella* sp.		Phosphate solubilizing bacteria	[173]
	*Pseudomonas peli*		Production of phytohormone (Indole acetic acid)	[172]
	*Arthrobacter methylotrophus* IARI-HHS1-25 *Bacillus pumilus* *Alcaligenes* sp.		ACC deaminase activity Siderophore production Biocontrol activity	[174]
3.	Mitigation of abiotic stress			
	*Trichoderma harzianum*	Xerophiles	Drought stress mitigation in rice genotypes	[175]
	*Pseudomonas* sp.	Halophiles	Salinity tolerance in *Triticum* *aestivum*	[26]
	*Pseudomonas aeruginosa* *Alcaligenes faecalis* *Proteus penneri*	Xerophiles	Enhanced protein, sugar, and water content in *Z. mays*	[122]
	*Paecilomyces formosus*	Thermophiles	Plant adaptation to heat stress Production of secondary metabolites	[129]
	*Arthrobacter* sp. *Burkholderia* sp. *Pseudomonas* sp.	Psychrophiles	PGP attributes Nutrition uptake Soil health maintenance	[176]
4.	*Pseudomonas stutzeri* *Streptomyces* sp. *Serratia marcescens*	----	Mitigation of biotic stress Tolerance to fungal phytopathogens	[177,178]
	*Trichoderma* sp. *Pseudomonas* sp.	----	Induce systemic resistance (ISR) against phytopathogens	[179,180]

## Data Availability

Not applicable.
